# Elucidation of Molecular Mechanisms of Streptozotocin-Induced Oxidative Stress, Apoptosis, and Mitochondrial Dysfunction in Rin-5F Pancreatic *β*-Cells

**DOI:** 10.1155/2017/7054272

**Published:** 2017-08-06

**Authors:** Arwa M. T. Al Nahdi, Annie John, Haider Raza

**Affiliations:** Department of Biochemistry, College of Medicine and Health Sciences (CMHS), UAE University, Al Ain, UAE

## Abstract

Streptozotocin is a pancreatic beta-cell-specific cytotoxin and is widely used to induce experimental type 1 diabetes in rodent models. The precise molecular mechanism of STZ cytotoxicity is however not clear. Studies have suggested that STZ is preferably absorbed by insulin-secreting *β*-cells and induces cytotoxicity by producing reactive oxygen species/reactive nitrogen species (ROS/RNS). In the present study, we have investigated the mechanism of cytotoxicity of STZ in insulin-secreting pancreatic cancer cells (Rin-5F) at different doses and time intervals. Cell viability, apoptosis, oxidative stress, and mitochondrial bioenergetics were studied. Our results showed that STZ induces alterations in glutathione homeostasis and inhibited the activities of the respiratory enzymes, resulting in inhibition of ATP synthesis. Apoptosis was observed in a dose- and time-dependent manner. Western blot analysis has also confirmed altered expression of oxidative stress markers (e.g., NOS and Nrf2), cell signaling kinases, apoptotic protein-like caspase-3, PARP, and mitochondrial specific proteins. These results suggest that STZ-induced cytotoxicity in pancreatic cells is mediated by an increase in oxidative stress, alterations in cellular metabolism, and mitochondrial dysfunction. This study may be significant in better understanding the mechanism of STZ-induced *β*-cell toxicity/resistance and the etiology of type 1 diabetes induction.

## 1. Introduction

Streptozotocin (STZ), [N-(methylnitrosocarbamoyl)-*α*-D-glucosamine], is a broad spectrum antibiotic derived from the bacterium *Streptomyces achromogenes* [1]. It is a DNA alkylating agent and is often used as an antibacterial as well as anticancer agent [2, 3]. However, it is not a preferred drug for the treatment of cancers. This is due to genotoxic effects which lead to drug resistance [4]. STZ is known to be a pancreatic beta-cell-specific cytotoxin and is therefore being widely used to induce experimental type 1 diabetes in rodent models [5, 6].

STZ is a glucose analogue that is selectively accumulated in pancreatic beta-cells via a GLUT 2 glucose transporter in the plasma membrane [7, 8]. STZ toxicity in beta-cells is dependent on GLUT 2 expression. Hosokawa and his colleagues revealed that in transgenic mice, GLUT 2-expressing beta-cells are sensitive to the toxic effects of STZ whereas GLUT 1-expressing islets are completely resistant [9]. After entering the beta-cells via the GLUT 2 transporter, it causes DNA damage due to the DNA alkylating activity of its methyl nitrosourea moiety [10, 11], which, in turn, results in DNA fragmentation [12]. Subsequently, the fragmented DNA activates poly (ADP-ribose) synthetase to repair DNA. Poly ADP-ribosylation leads to the depletion of cellular NAD+ and ATP [12, 13]. The decreased ATP synthesis is demonstrated by dephosphorylation which provides more substrates for xanthine oxidase, resulting in the formation of hydrogen peroxide and hydroxyl radicals [14, 15] causing oxidative stress. Furthermore, the presence of N-methyl-N-nitrosourea side chain has the ability to release nitric oxide [16, 17] that inhibits aconitase activity, resulting in mitochondrial dysfunction. STZ is diabetogenic due to its targeted GLUT 2-dependent action in the pancreatic *β*-cells. The exact mechanism of cytotoxicity is still not clear. However, both apoptotic and necrotic cell deaths of *β*-cells have been reported. The cytotoxicity of STZ is presumed to be mediated by reactive oxygen species (ROS), reactive nitric oxide species (NO/RNS), and induction of inflammatory responses [16, 17]. Using both in vitro cell culture and in vivo diabetic rodent models for STZ-induced toxicity, we have demonstrated that STZ induces cellular oxidative stress and mitochondrial respiratory dysfunction [18–20].

In the present study, we have further investigated the mechanism of STZ cytotoxicity on insulin-secreting Rin-5F cells. Our results demonstrate that the effects of STZ are dose and time dependent, causing oxidative stress-associated alterations in GSH redox metabolism and mitochondrial respiratory dysfunction leading to increased apoptosis in Rin-5F cells. We have also identified some of the key apoptotic and oxidative stress molecular markers which exhibit altered expression in STZ-treated Rin-5F cells. In addition, we have also demonstrated that STZ treatment has induced the activities of CYP1A2 and CYP1A1 suggesting their potential role in STZ metabolism. These results may be significant in understanding the mechanism of STZ-induced *β*-cell cytotoxicity/apoptosis and the ability of pancreatic cells to metabolize other xenobiotics in oxidative stress conditions.

## 2. Materials and Methods

### 2.1. Materials

Streptozotocin (STZ), reduced and oxidized glutathione (GSH/GSSG), 1-chloro 2,4-dinitrobenzene (CDNB), cumene hydroperoxide, glutathione reductase, 3-(4,5-dimethylthiazol-2-yl)-2,5-diphenyltetrazolium bromide (MTT), NADH, NADPH, cytochrome c, coenzyme Q2, sodium succinate, antimycin A, dodecyl maltoside, resorufin, 7-ethoxyresorufin, methoxyresorufin, Hoechst 33342, and ATP bioluminescent somatic cell assay kits were purchased from Sigma-Aldrich (St. Louis, MO, USA). 2′,7′-Dichlorofluorescein diacetate (DCFDA) was procured from molecular probes (Eugene, OR, USA). Kits for nitric oxide and caspase-3 and caspase-9 assays were purchased from R&D Systems Inc., MN, USA, and that for lipid peroxidation (LPO) from Oxis International Inc. (CA, USA). Kits for GSH/GSSG assay were procured from Promega Corp. (Madison, WI, USA). Apoptosis detection kits for flow cytometry were purchased from BD Pharmingen (BD Biosciences, San Jose, USA). Rin-5F cells were obtained from American Type Culture Collection (Manassas, VA, USA). Polyclonal antibodies against beta-actin, caspase-3, PARP, NOS-2, Nrf2, GLUT 2, Bax, Bcl-2, Akt, and p-Akt were purchased from Santa Cruz Biotechnology Inc. (Santa Cruz, CA, USA). Reagents for cell culture, SDS-PAGE, and Western blot analyses were purchased from Gibco BRL (Grand Island, NY, USA) and Bio-Rad Laboratories (Richmond, CA, USA).

### 2.2. Cell Culture and Treatment

Rin-5F cells were grown in poly-L-lysine-coated 75 cm^2^ flasks (~2.0–2.5 × 10^6^ cells/mL) in RPMI1640 medium supplemented with 1% nonessential amino acids, 2 mM glutamine, and 10% heat-inactivated fetal bovine serum in a humidified incubator in the presence of 5%–95% CO_2_ air at 37°C. Cells were treated with different concentrations of STZ (0–10 mM) dissolved in citrate buffer, pH 4.4, and diluted in RPMI1640 to appropriate concentrations just before use for different time intervals (24 h–48 h). Control cells were treated with vehicle alone. Concentrations and time points for STZ treatment in this study were based on MTT cytotoxicity tests and previously published reports [18, 21]. After the desired time of treatment, cells were harvested, washed with PBS (pH 7.4), and homogenized in H-medium buffer (70 mM sucrose, 220 mM mannitol, 2.5 mM HEPES, 2 mM EDTA, and 0.1 mM phenylmethylsulfonylfluoride, pH 7.4) at 4°C. Mitochondrial and postmitochondrial fractions were then isolated by differential centrifugation. Cellular fractionation to prepare mitochondria fractions was performed by centrifugation, and the purity of the isolated fractions for cross contaminations was checked as described previously [20]. Protein concentration was determined by the Bradford method [22].

### 2.3. MTT Cell Viability Test

Mitochondrial dehydrogenase-based cell viability test in 96-well plates (~2 × 10^4^ cells/well) was assayed by MTT conversion to formazan after treatment with different concentrations (0–10 mM) of STZ for different time intervals (24 h–48 h). The viable cells were quantitated using an ELISA reader (Anthos Laboratories, Salzburg, Germany) at 550 nm after subtracting the appropriate control value.

### 2.4. Measurement of Reactive Oxygen Species (ROS), NO, and LPO

Intracellular production of reactive oxygen species was measured using the cell permeable probe, DCFDA. Briefly, STZ-treated and control cells (~1 × 10^5^ cells/mL) were grown on cover slips and incubated with 5 *μ*M DCFDA for 30 min at 37°C. Cells were washed twice with PBS, and fluorescence was immediately visualized using the Olympus fluorescence microscope. DCFDA-based ROS assay was also performed and measured fluorimetrically as described before [18].

For NO assay, Rin-5F cells (2 × 10^5^ cells/well) were cultured in petri plates for 24 h prior to STZ treatments. NO production was determined by measuring the concentration of total nitrite in the culture supernatants using Griess reagent (R&D Systems Inc.).

LPO in the cell extracts of STZ-treated and control Rin-5F cells was measured using the LPO-586 kit according to the manufacturer's recommended protocol and the concentration of MDA calculated from the standard curve.

### 2.5. Apoptosis Measurement after STZ Treatments

#### 2.5.1. Nuclear Staining with Hoechst33342

Apoptosis measurement was performed by Hoechst dye staining of fragmented nuclei. Cover slips with adherent cells were treated with STZ, and cells were fixed with 3.7% formaldehyde and stained with Hoechst33342 (10 *μ*g/mL) for 20 min at room temperature. The cover slips were washed, mounted on glass slides, and analyzed by fluorescence microscopy. Cells with signs of apoptosis showed fragmented nuclei.

#### 2.5.2. Flow Cytometry

The apoptosis assay using flow cytometry was performed according to the vendor's protocol (BD Pharmingen, BD Biosciences, San Jose, USA) as described before [23]. Briefly, treated and control untreated cells were trypsinized, washed in PBS, and resuspended (1 × 10^6^ cells/mL) in binding buffer (10 mM HEPES, pH 7.4, 140 mM NaCl, 2.5 mM CaCl_2_). A fraction (100 *μ*L/1 × 10^5^ cells) of the cell suspension was incubated with 5 *μ*L annexin V conjugated to FITC and 5 *μ*L propidium iodide (PI) for 15 min at 25°C in the dark. 400 *μ*L of binding buffer was added to the suspension, and apoptosis was measured immediately using a Becton Dickinson FACScan analyzer. The apoptotic cells were estimated by the percentage of cells that were stained positive for annexin V-FITC while remaining impermeable to PI (AV+/PI−). This method was also able to distinguish viable cells (AV−/PI−) and cells undergoing necrosis (AV+/PI+).

#### 2.5.3. Assay of Caspase Activities

Caspase-3 and caspase-9 activities were measured in the cell lysate using detection kits as per the vendor's protocol. The assay is based on spectrophotometric detection of the chromophore p-nitroanilide (pNA) after cleavage from the labeled substrates, DEVD-pNA, and LEHD-pNA to measure the activities of caspase-3 and caspase-9, respectively. The pNA light emission was then quantified using a microtiter plate reader at 405 nm.

### 2.6. SDS-PAGE and Western Blot Analysis

Proteins from cell extracts (30 *μ*g) from control and STZ-treated cells were separated electrophoretically on 12% SDS-PAGE [24] and transferred on to nitrocellulose paper by Western blotting [25]. Transferred proteins were probed with primary antibodies against caspase-3, PARP, Akt, p-Akt, NOS-2, Nrf2, Bax, Bcl-2, and GLUT 2. Immunoreactive bands were visualized using the appropriate conjugated secondary antibodies. Equal loading of protein was confirmed using beta-actin as the loading control. After the development of the blots, the bands were visualized and further densitometric analysis was performed using the Typhoon FLA 9500 system (GE Healthcare, Uppsala, Sweden) and expressed as relative ratios normalized against actin or other proteins as appropriate.

### 2.7. Measurement of CYP 450-Dependent Enzyme Activities

CYP1A1 and CYP1A2 activities in the microsomal fraction from treated and untreated control cells were measured spectrofluorometrically using 7-ethoxyresorufin and methoxyresorufin, respectively, as substrates [26, 27] by standard methods as described before [28–30].

### 2.8. Measurement of GSH Metabolism

Rin-5Fcells were treated with different doses of STZ for different time intervals as mentioned above. GSH/GGSG ratios and activities of GSH-Px and glutathione S-transferase (GST) were measured in the STZ-treated and STZ-untreated control cell extracts. GSH/GGSG ratios were measured using the GSH/GGSG-Glo kit as per the vendor's protocol. Briefly, STZ-treated and STZ-untreated control cells were lysed with either total or oxidized glutathione reagent. For the oxidized glutathione measurement, the total GSH was blocked using NEM reagent and the oxidized glutathione was reduced. The total reduced glutathione then converts a specific probe, luciferin-NT to luciferin in the presence of a GST enzyme coupled to firefly luciferase. The luciferin formed gives a luminescent signal, which is proportional to the amount of GSH. The total glutathione and oxidized glutathione are then measured from the standard curve, and the GSH/GSSG ratios calculated. GST activity using CDNB [31] and GSH-Px activity using cumene hydroperoxide [32] as substrates were measured by standard protocols as described before [33–36].

### 2.9. Measurement of Activities of Mitochondrial Respiratory Enzyme Complexes and ATP Content

Cell extracts (5 *μ*g protein) from STZ-treated and STZ-untreated control Rin-5F cells were suspended in 1.0 mL of 20 mM KPi buffer, pH 7.4, in the presence of the detergent, lauryl maltoside (0.2%). NADH ubiquinone oxidoreductase (complex I), succinate-cytochrome c reductase (complex II/III), and cytochrome c oxidase (complex IV) were measured using the substrates coenzyme Q2, succinate-cytochrome c, and reduced cytochrome c, respectively, by the methods of Birch-Machin and Turnbull [37] as described before [35, 36]. The ATP content in control and STZ-treated cells was determined using the ATP bioluminescent cell assay kit according to the manufacturer's suggestion (Sigma-Aldrich, St Louis, MO), and samples were read using the TD-20/20 luminometer (Turner Designs, Sunnyvale, CA).

### 2.10. Statistical Analysis

Values shown are expressed as mean ± SEM of three individual experiments. Statistical significance of the data was assessed using SPSS software (version 23) by analysis of variance followed by Dunnett's post hoc analysis. *P* values ≤ 0.05 were considered statistically significant.

## 3. Results

### 3.1. Effect of STZ on Rin-5F Cell Morphology and Viability

**Figure 1:**
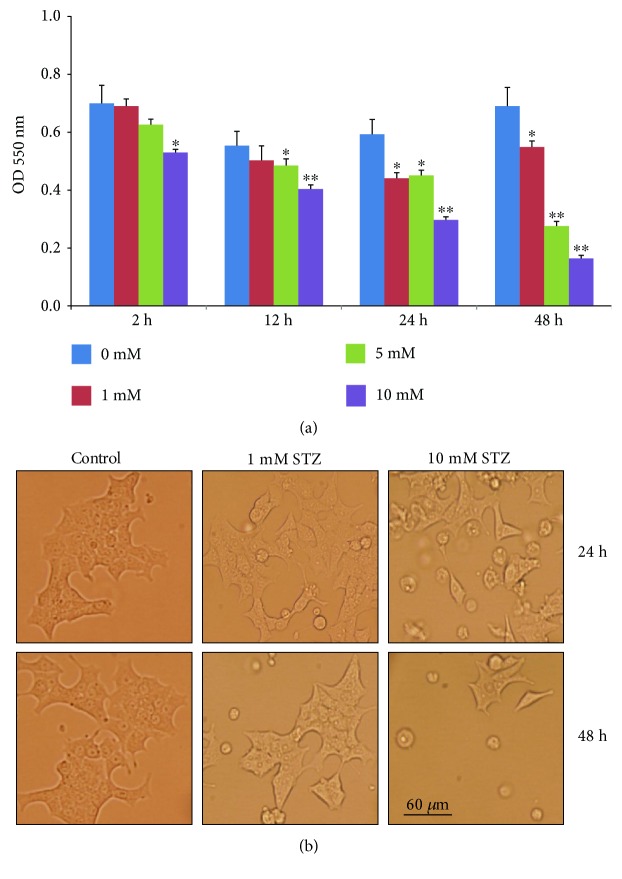
MTT cell viability assay and morphology of cells after STZ treatment. Rin-5F cells (~2 × 10^4^) were grown in 96-well plates for 24 h and treated with different concentrations (0–10 mM) of STZ for different time intervals. The formazan crystals formed, following the reduction of MTT by metabolically active (viable) cells, were solubilized in acidified isopropanol and quantitated using the ELISA reader at 550 nm (a). Results are expressed as mean ± SEM for three experiments. Asterisks indicate significant difference (^∗^*p* ≤ 0.05, ^∗∗^*p* ≤ 0.005) relative to the untreated control cells. The morphological integrity of the STZ-treated and STZ-untreated control cells was also checked and photographed (20x) under a light microscope (b).

A decrease in mitochondrial dehydrogenase-based cell survival was observed only with higher concentrations of STZ after 2–12 h (Figure 1(a)). Significant alterations in cell viability were observed even at low concentration after 24–48 h treatments. The maximum inhibition (60–70%) was observed in cells treated with 10 mM STZ for 24 h and 48 h. Since significant alterations in cell viability were observed at 24 h and 48 h, with minimal toxicity using 1 mM STZ and maximal toxicity using 10 mM STZ, these two time points and concentrations were used in our further studies to elucidate the mechanism of STZ toxicity.

Figure 1(b) shows the morphology of control untreated Rin-5F cells as well as cells treated with different doses of STZ at different time intervals. As seen in the figure, after STZ treatment, the normal flattened cells tend to round off, losing their normal morphology. When the cells were treated with 10 mM STZ for 48 h, the rounded cells started detaching from the plate, indicating increased cell death.

### 3.2. Effect of STZ on Oxidative Stress

**Figure 2:**
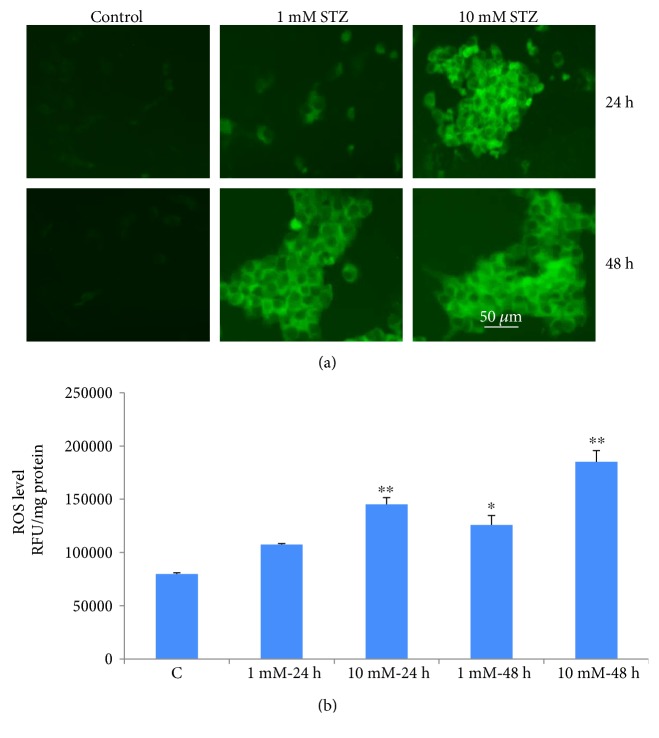
ROS production in STZ-induced cells. Intracellular production of reactive oxygen species was measured in control untreated and STZ-treated Rin-5F cells with different concentrations (0–10 mM) for different time intervals, using the cell permeable probe, DCFDA. Cells (~1 × 10^5^ cells/mL) were grown on cover slips and incubated with 5 *μ*M DCFDA for 30 min at 37°C. Cells were washed twice with PBS, and fluorescence was immediately visualized using an Olympus fluorescence microscope. Representative slides from untreated control and STZ-treated cells from three experiments are shown (a). Original magnification ×200. Production of reactive oxygen species was also measured fluorimetrically in control untreated and STZ-treated cells (b). Results are expressed as mean ± SEM of three experiments. Asterisks indicate significant difference (^∗^*p* ≤ 0.05, ^∗∗^*p* ≤ 0.005) relative to the untreated control cells.

Increased ROS production in Rin-5F cells treated with different doses of STZ at different time intervals was captured microscopically using the probe, DCFDA, which measures the overall ROS production. Maximum fluorescence was observed with 10 mM STZ at 24 h and 48 h (Figure 2(a)). A time- and dose-dependent increase in intracellular ROS production was also measured fluorometrically as shown in Figure 2(b). Significant increases in ROS production were observed, with a marked increase (2-fold and 3-fold) observed with 10 mM STZ at 24 h and 48 h, respectively.

**Figure 3:**
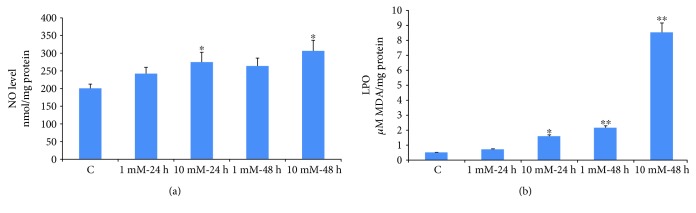
NO production and lipid peroxidation in STZ-induced cells. NO production was determined by measuring the concentration of total nitrite in the culture supernatants (a) with Griess reagent (R&D Systems Inc.). Lipid peroxidation (LPO) in the control and STZ-treated cells was measured as total amount of malondialdehyde (b) as per the vendor's protocol (Oxis Research Inc.). Results are expressed as mean ± SEM of three experiments. Asterisks indicate significant difference (^∗^*p* ≤ 0.05, ^∗∗^*p* ≤ 0.005) relative to the untreated control cells.

NO production was significantly increased (25–40%) in Rin-5F cells treated with 10 mM STZ for 24 or 48 h (Figure 3(a)) whereas a marginal increase was observed with 1 mM STZ treatment after 48 h.

In parallel to ROS production, LPO was significantly increased in a dose- and time-dependent manner after treatment with STZ (Figure 3(b)). Treatment with 10 mM STZ for 48 h had markedly increased the production of malondialdehyde (MDA). These results clearly indicate the increased oxidative stress in Rin-5Fcells treated with STZ.

### 3.3. Effects of STZ on Cell Survival and Apoptosis

**Figure 4:**
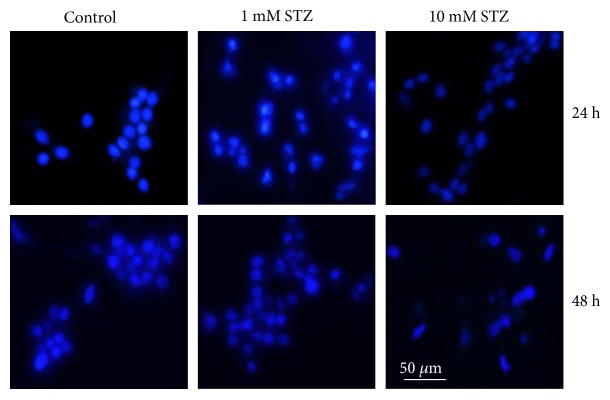
STZ-induced DNA fragmentation. Staining of fragmented nuclei of STZ-treated and STZ-untreated cells was performed by using Hoechst33342 dye. Cover slips with adherent cells were treated with STZ, fixed with 3.7% formaldehyde, and stained with Hoechst33342 (10 *μ*g/mL) for 20 min at room temperature. The cover slips were washed, mounted on glass slides, and analyzed by fluorescence microscopy. Cells with signs of apoptosis showed fragmented nuclei. Representative slides from three experiments are shown. Original magnification ×200.

STZ induced time- and dose-dependent apoptosis in Rin-5F cells as detected by an increase in nuclear condensation was observed by Hoechst staining (Figure 4).

**Figure 5:**
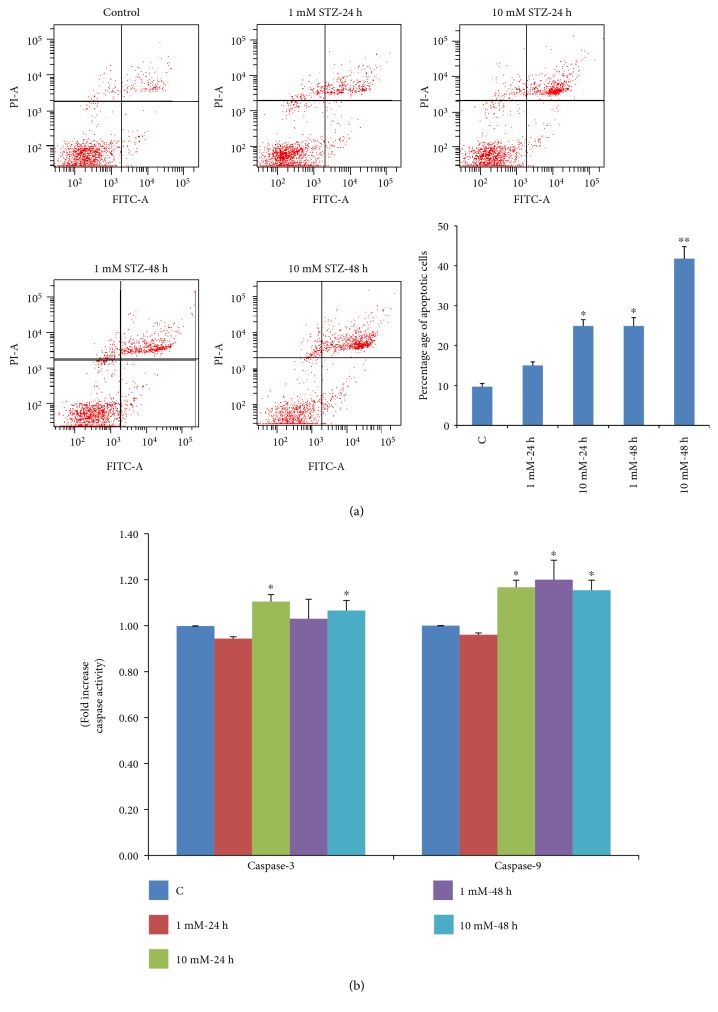
STZ-induced apoptosis. Apoptosis was measured in Rin-5F cells treated with different doses of STZ at different time intervals by flow cytometry using FACSDiva software. Representative dot plots are shown, and percentage of apoptotic cells is represented as a histogram (a). Activity of caspases was measured in cells (b) treated with different doses of STZ at different time intervals colorimetrically using the respective substrates as described in the vendor's protocol (R&D Systems Inc.). Results are expressed as mean ± SEM of three experiments. Asterisks indicate significant difference (^∗^*p* ≤ 0.05, ^∗∗^*p* ≤ 0.001) relative to the untreated control cells.

Figure 5(a) shows a significant increase in the percentage of cells undergoing early/late apoptosis by increasing the time and dose of STZ treatment. Treatment of Rin-5F cells with 1 mM STZ for 24 h caused 12% of cells to go into late apoptosis, which further increased to 22% at 10 mM STZ. Moreover, increasing the time of STZ treatment caused a further increase in the late apoptotic cells (almost 20% to 36% at 1 mM and 10 mM, respectively). The histogram in Figure 5(a) represents the percentage of total apoptotic cells after treatment with STZ at different concentrations and time intervals. This increase in apoptosis was also confirmed by increased activities of caspase isoenzymes (3 and 9) (Figure 5(b)). The activity of intrinsic apoptotic enzyme caspase-9 significantly increased with increasing time and dose of STZ treatment. However, the activities of terminal apoptotic enzyme caspase-3 showed significant increase after treatment with 10 mM STZ at 24 h and 48 h.

### 3.4. Effect of STZ on the Expression of Apoptotic Marker Proteins

**Figure 6:**
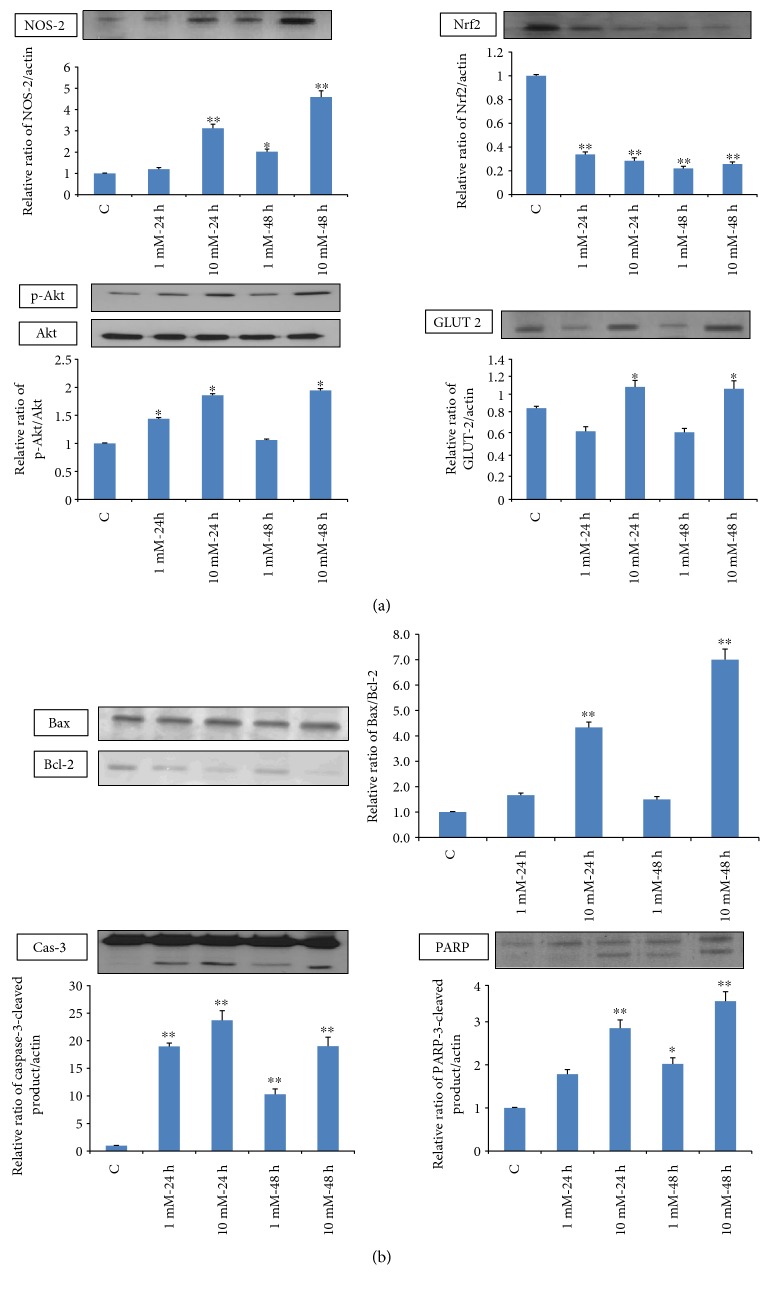
Expression of apoptotic protein markers. Total extracts (30 *μ*g protein) from control and Rin-5F cells treated with different doses of STZ at different time intervals were separated on 12% SDS-PAGE and transferred on to nitrocellulose paper by Western blotting. NOS-2 Nrf2, Akt, p-Akt, and GLUT 2 (a) and caspase-3, PARP, Bax, and Bcl-2 proteins (b) were detected using specific antibodies against these proteins. Beta-actin was used as a loading control. The quantitation of proteins bands is expressed as relative ratios normalized against actin or other proteins as appropriate. The figures are representative of three experiments. Asterisks indicate significant difference (^∗^*p* < 0.05, ^∗∗^*p* < 0.005) relative to the untreated control cells.

Figure 6(a) shows alterations in the expression of oxidative stress marker proteins, NOS-2 and Nrf2. In increased phosphorylation of the cell signaling kinase, Akt was observed at high doses of STZ. A mild increase in GLUT 2 expression was observed suggesting increased STZ/glucose uptake through this mechanism.  Figure 6(b) shows a marked cleavage of apoptotic marker protein, caspase-3, as well as PARP and alterations in the expression of intrinsic mitochondrial-specific proteins like Bcl-2 and Bax which were also observed at high doses. All of these results confirm the increased oxidative stress observed in these cells after STZ treatment.

### 3.5. Effects of STZ on CYP 450 Activities

**Figure 7:**
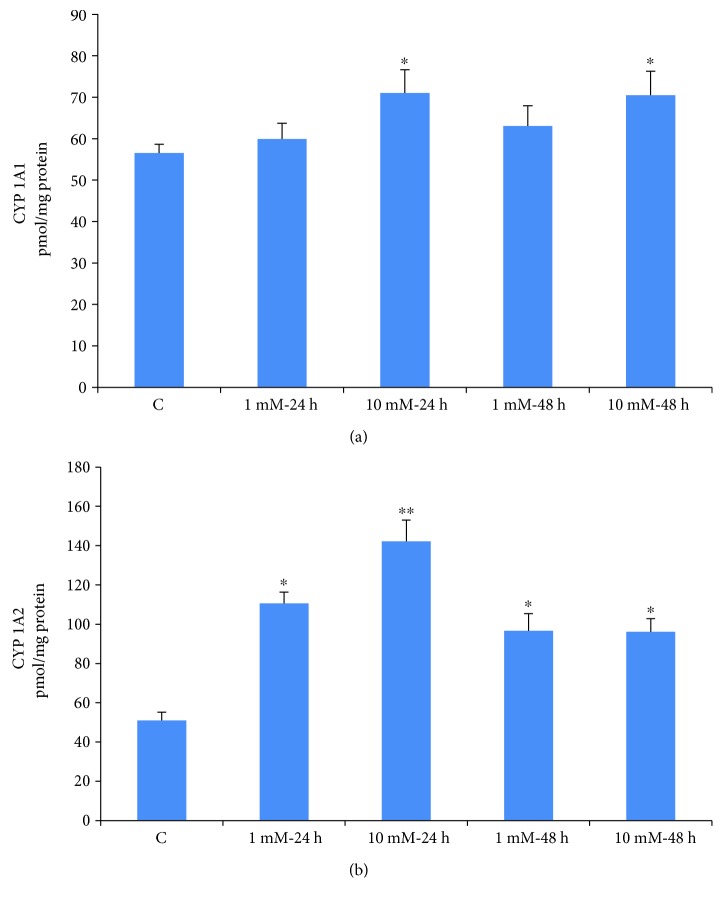
STZ-induced alterations in CYP activities. CYP 1A1 and CYP 1A2 activities were measured in Rin-5F cells treated with different doses of STZ at different time intervals using the respective substrates as described in the Materials and Methods. Results are expressed as mean ± SEM of three experiments. Asterisks indicate significant difference (^∗^*p* ≤ 0.05, ^∗∗^*p* ≤ 0.001) relative to the untreated control cells.

Isoenzyme-specific substrates were used to measure the microsomal activities of CYP1A1 and CYP1A2 in Rin-5F cells treated with STZ at different doses and time intervals. CYP1A1 activity showed significant increase with 10 mM STZ at 24 h and 48 h (Figure 7(a)) while no significant increase of enzyme activity was observed with 1 mM STZ. CYP1A2 activity, on the other hand, increased significantly (2-3 fold) at all doses and time points (Figure 7(b)). These results may suggest the involvement of CYP1A family of isoenzymes in STZ metabolism.

### 3.6. Effects of STZ on the GSH/GSSG Ratio and GSH Metabolism

**Figure 8:**
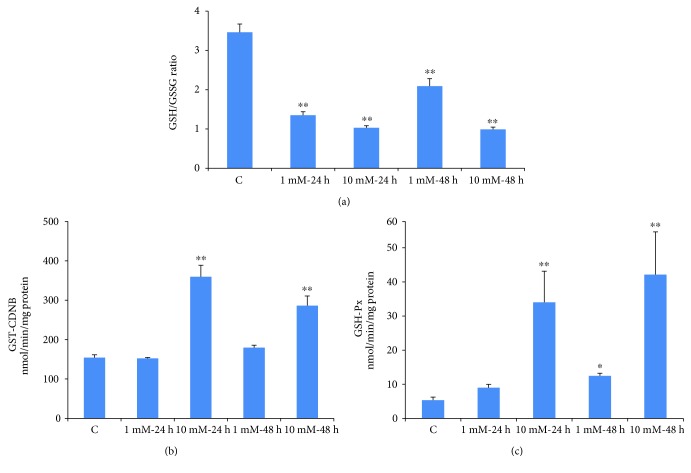
STZ-induced alterations in GSH metabolism. Rin-5F cells were treated with different doses of STZ for different time intervals. GSH/GSSG ratio (a), GST (b), and GSH-Px (c) were measured. Results are expressed as mean ± SEM of three experiments. Asterisks indicate significant difference (^∗^*p* ≤ 0.05, ^∗∗^*p* ≤ 0.005) relative to the untreated control cells.

Dose- and time-dependent decrease (60–70%) in the ratio of cellularly reduced GSH and oxidized GSSG was observed after STZ treatment (Figure 8(a)). However, a slight recovery in the GSH/GSSG ratio after 48 h of treatment with lower dose of STZ suggests some delay in recycling of oxidized GSSG.

GSH-conjugating enzyme, GST, was however significantly increased (almost 2 fold) at high doses of STZ (Figure 8(b)). On the other hand, a marginal increase was observed with 1 mM STZ at 48 h. A marked increase (about 6–8 fold) in GSH-Px activity was also observed when cells were treated with 10 mM STZ both at 24 h and 48 h (Figure 8(c)). Though statistically not significant, a marginal increase in enzyme activity was also observed in cells treated with 1 mM STZ for 24 h. These results may suggest an increased cellular GSH conjugation and detoxification mechanism as an adaptation towards STZ metabolism and toxicity.

### 3.7. Effects of STZ on Mitochondrial Respiratory Function and ATP Production

**Figure 9:**
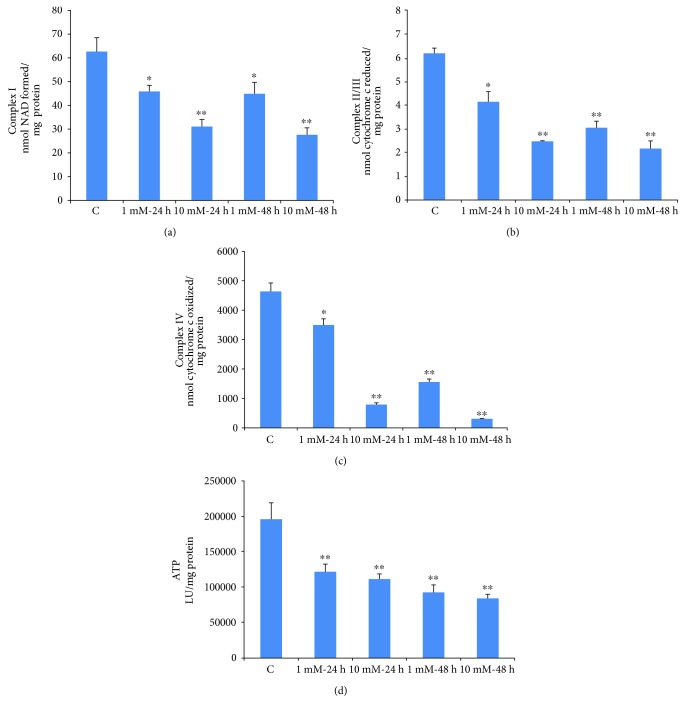
STZ-induced alterations in mitochondrial enzyme activity. Rin-5F cells were treated with different doses of STZ for different time intervals. Respiratory complex I (a) complex II/II (b), and complex IV (c) were measured using their respective substrates as described in the Materials and Methods. ATP content (d) was measured using the ATP bioluminescent somatic cell assay kit. Results are expressed as mean ± SEM of three experiments. Asterisks indicate significant difference (^∗^*p* ≤ 0.05, ^∗∗^*p* ≤ 0.001) relative to the untreated control cells.

Figure 9 shows the effects of STZ treatment on mitochondrial respiratory enzymes and bioenergetics. Both 1 mM and 10 mM STZ caused a significant inhibition (40–50%) in NADH ubiquinone oxidoreductase (complex I) enzyme activity after 24 h and 48 h (Figure 9(a)). The activity of succinate-cytochrome c reductase (complex II/III) was also significantly inhibited (40–65%) after 24 h and 48 h, with both 1 mM and 10 mM STZ (Figure 9(b)). On the other hand, the activity of the terminal respiratory enzyme complex IV was markedly inhibited (6–8 fold) by increasing the dose and time of STZ treatment (Figure 9(c)). Consistent to the reduction in mitochondrial respiratory activity, ATP levels were also markedly decreased by increasing the dose and the time of STZ treatment (Figure 9(d)).

### 3.8. The Mechanism of STZ-Induced Cytotoxicity in Rin-5F Cells

**Figure 10:**
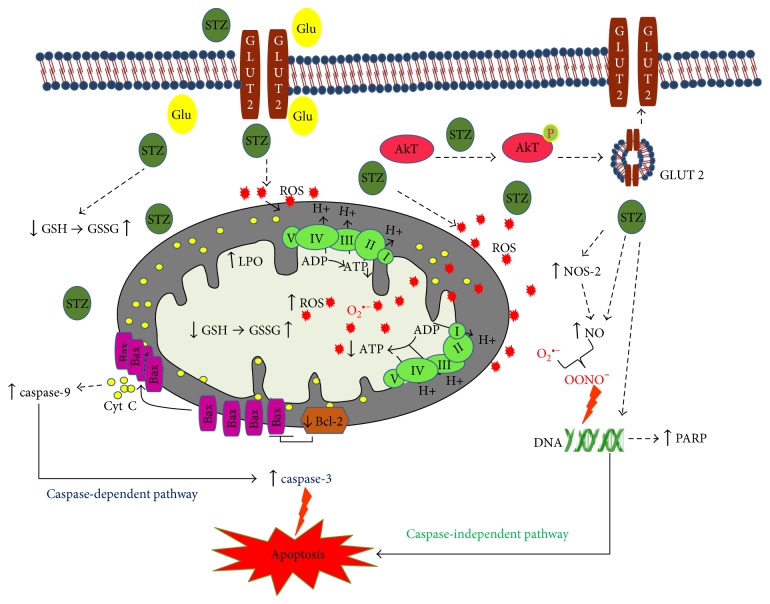
Schematic model depicting the mechanism of STZ-induced cytotoxicity in Rin-5F cells. STZ competes with glucose (Glu) to enter the cells via GLUT 2 receptors, causing Akt phosphorylation, which, in turn, causes further translocation of the GLUT 2 receptors. The model also shows that STZ induces cytotoxicity and apoptosis by increased ROS/NOS production, oxidative/nitrosative stress, increased LPO, DNA damage, a decreased GSH/GSSG ratio, and mitochondrial dysfunction. Upward arrows (↑) indicate increase and downward arrows (↓) indicate decrease.

Figure 10 shows a schematic model depicting the mechanisms of STZ-induced cytotoxicity in Rin-5F cells. STZ competes with glucose to enter the cells via GLUT 2 receptors, causing Akt phosphorylation, which in turn, causes further translocation of the GLUT 2 receptors. STZ-induced cytotoxicity increased ROS/NOS production, LPO, and DNA damage and decreased the GSH/GSSG ratio. Moreover, STZ induced mitochondrial dysfunction through inhibition of the activities of the mitochondrial respiratory enzymes, complex I, complex II/III, and complex IV, and decreased ATP production. STZ treatment induced apoptosis by both caspase-dependent pathway through the activation of caspase-3 and caspase-9 and caspase-independent pathway by DNA fragmentation and PARP activation.

## 4. Discussion

Pancreatic *β*-cell cytotoxicity has been observed even at therapeutic doses (up to 15 mM) of STZ when used as an antineoplastic drug for different types of cancer, and this level of STZ induces apoptosis in pancreatic *β*-cells [10, 15, 38, 39]. Recent studies have shown that STZ is also toxic to neuroendocrine cells of the gut [40] as well as other GLUT 2-expressing organs such as the kidneys, liver, and brain [41]. It has been reported that single intracerebroventricular STZ injection chronically decreases glucose uptake and produces effects that resemble features of Alzheimer's disease [41]. Since the cellular uptake of STZ competes with glucose uptake and is considered to be dependent upon the specific expression of selective Glut transporters [9, 42], the differential cytotoxicity by STZ in different cellular systems may be associated with the selective uptake of STZ, its metabolic activation, and detoxification in specific cell types as well as on the redox homeostasis and mitochondrial bioenergetics in these cells [14, 43]. We have previously demonstrated that STZ induces oxidative stress and mitochondrial respiratory dysfunction not only in the pancreas but also in the liver, kidney, and other organs [20, 44]. We have also shown that offspring born to STZ-treated diabetic mother rats also exhibited oxidative stress-associated complications in different organs [19]. We have previously reported STZ-induced apoptotic cell death, oxidative stress, and mitochondrial dysfunction in HepG2 cells [18]. Oxidative/nitrosative stress and alterations in mitochondrial function and NF-kB-dependent apoptosis were also reported when HepG2 cells were treated with STZ [18]. Our present study has been further extended to elucidate the mechanism of STZ-induced oxidative stress, alterations in respiratory function, and identification of apoptotic markers in Rin-5F cells treated at different doses and time points. The selection of time points and doses of STZ was based on the alterations in cell viability and morphology (as shown in Figures 1(a) and 1(b)) and also based on our previous studies on HepG2 cells where treatment at lower doses for short time periods had minimum effects on cell viability [18]. Our present results on STZ-treated Rin-5F cells have clearly indicated the increase in ROS/RNS production, increased lipid peroxidation, increased expression of oxidative stress marker protein NOS-2, inhibition in GSH synthesis, and alteration in GSH metabolism by GST and GSH–Px. Increased activities of GST and GSH-Px suggest that STZ-induced oxidative stress triggers the activation of antioxidant defensive mechanism to protect the beta-cell death. We also observed marked activation of CYP1A2 activity and moderate activation of CYP1A1 activity in STZ-treated Rin-5F cells, probably suggesting the metabolism of STZ by the arylhydrocarbon receptor- (Ahr-) activated CYP1 enzymes. We have previously reported increased expression of CYP isoenzymes in STZ-treated type 1 and type 2 diabetic models [19, 36, 45, 46]. However, decreased expression of the antioxidant responsive protein, Nrf2, was observed after STZ treatment, which decreased drastically after 48 h. Pancreatic cells contain very low levels of antioxidant enzymes; thus, these cells are particularly sensitive to oxidative stress [47]. Nrf2 is considered a master regulator of the antioxidant response, and decreased expression of Nrf2 by STZ has been shown by various researchers [48, 49]. The reason for this loss of Nrf2 expression in pancreatic cells could be due to the STZ-induced increased intracellular ROS and oxidized-to-reduced GSH ratio which has been reported in this study as well as by other researchers. Glutathione transferase (GST) is a detoxifying enzyme that plays a protective role against oxidative stress. The induction of this family of enzymes is thought to be an adaptive response to chemical toxicity and oxidative stress within cells. In addition to Nrf2, GST induction is under the regulation of the “AhR gene battery” as well as other transcription factors which are activated during chemical detoxification and oxidative stress with increased ROS production. Reports also suggest that the induction of AhR-regulated enzymes, CYP1A1 and 1A2, by xenobiotics also induces various forms of GSTs for their metabolic detoxifications [50–52]. Our present study also demonstrates that the activities of both CYP1A1/1A2 and GST enzymes have been activated after STZ treatment in Rin-5F cells, suggesting the involvement of AhR-dependent activation of CYP1A1/1A2 and GSTs. It has been shown that Nrf2 protein upregulates the antiapoptotic protein, Bcl-2, along with a battery of other cytoprotective proteins and enzymes and prevents cellular apoptosis [53]. In our study, we observed a dose-dependent decrease in Bcl-2 expression with a concomitant increase in proapoptotic protein, Bax, and increased cleavage of caspase-3. This could be due to the reduced expression of Nrf2 protein. A dose-dependent increase in PARP cleavage was also observed, which supported our observation on fragmentation of DNA at high doses.

Like HepG2 cells [18], the STZ-treated Rin-5F cells also exhibited mitochondrial dysfunction followed by apoptosis. Activities of mitochondrial respiratory enzymes complex I, complex II/III, and complex IV were significantly inhibited, though the inhibition of complex IV was more pronounced than those of the other complexes, as some recovery in the enzyme activity was seen when cells were treated with 1 mM STZ for 48 h. As expected under these conditions, ATP level was also reduced in STZ-treated cells in a dose- and time-dependent manner. Increased oxidative stress and mitochondrial dysfunction resulted in increased apoptosis in STZ-treated Rin-5F cells. More apoptotic cell death was observed with high concentration of STZ (10 mM) for longer duration (48 h) compared to 1 mM STZ for 24 h (Figures 4 and 5). DNA fragmentation and activation of caspase-3 and caspase-9 confirmed the increased apoptosis after STZ treatment. Thus, the decrease in mitochondrial ATP synthesis and inhibition of respiratory enzymes, increased ROS/RNS production, lipid peroxidation and DNA fragmentation, and increased apoptosis have further confirmed and supported our previous studies as well as numerous other reports on the mechanism of STZ-induced cytotoxicity, in various in vivo and in vitro models, particularly in insulin-secreting pancreatic Rin-5F cells [14, 18, 19, 54–56]. Furthermore, our present study has also identified the increased expression of molecular oxidative stress and apoptotic marker such as NOS-2, as well as cleavage of caspase-3 and PARP in STZ-treated Rin-5F cells. In addition, our study also showed that STZ increases the phosphorylation of prosurvival protein, Akt, suggesting a role in altering insulin signaling and GLUT expression. It is possible that STZ stimulates Akt phosphorylation in Rin-5F cells to increase GLUT 2 transport to the membrane for the transport of STZ itself and to protect the cells from further oxidative/metabolic insult. Some studies have reported a corelation between Akt phosphorylation and GLUT 2 expression and translocation. Their reports suggest that expression and membrane translocation of GLUT 2 are substantially reduced in Akt knockout mice [57]. Our study has also confirmed increased GLUT 2 expression with increased Akt phosphorylation at high doses of STZ treatment.

Our results also support the observation reported that STZ induces cell resistance in *β*-cells towards its own toxicity. One explanation for this could be that, in addition to the other mechanisms as reported, the STZ-treated cell might be activating the prosurvival signals (e.g., Akt, as observed in this study) and increasing GLUT 2 levels, thus modulating glucose metabolism and uptake. Another reason could be the increased GST/GSH-Px-dependent detoxification processes in *β*-cells in order to defend cells from the deleterious effects of STZ and ROS [58, 59]. The altered expression of redox-sensitive protein, Nrf2, could also be a line of defense to protect the cells from the cytotoxic effects of STZ. A recent study has shown that cellular GST acts as a reservoir for NO and thus scavenges NO and detoxifies ROS [60] via GSH conjugation. This along with the increased GST/GSH-Px-dependent efflux/detoxification of STZ could render increased resistance to the cells against ROS/NO cytotoxicity.

## 5. Conclusion

In summary, here we provide additional evidence and have confirmed that the mechanism of STZ-induced cytotoxicity and apoptosis in Rin-5F cells is mediated by increased oxidative/nitrosative stress, mitochondrial dysfunction, and alterations in cell signaling. In addition, our results also suggest that STZ-treated Rin-5F cells also induces some cellular protection pathways as indicated by altered cell signaling and detoxification mechanisms which might be associated with the development of cellular resistance towards STZ. These results may be significant in better understanding the etiological mechanisms involved in STZ-induced toxicity/resistance in pancreatic as well as in other cellular systems.

## Abbreviations

STZ: Streptozotocin
MTT: 3-(4,5-Dimethylthiazol-2-yl)-2,5-diphenyltetrazolium bromide
ROS: Reactive oxygen species
NO: Nitric oxide
LPO: Lipid peroxidation
GSH: Glutathione
GST: Glutathione S-transferase
GSH-Px: Glutathione peroxidase
Nrf2: Nuclear factor erythroid 2-related factor 2
CDNB: 1-Chloro 2,4-dinitrobenzene
DCFDA: 2′,7′-Dichlorofluorescein diacetate
SDS-PAGE: Sodium dodecyl sulfate polyacrylamide gel electrophoresis.
